# Vpu Matchmakers as a Therapeutic Strategy for HIV Infection

**DOI:** 10.1371/journal.ppat.1000246

**Published:** 2009-05-29

**Authors:** Mauricio Montal

**Affiliations:** Section of Neurobiology, Division of Biological Sciences, University of California San Diego, La Jolla, California, United States of America; The Scripps Research Institute, United States of America

## Background and Significance

Chemoprophylaxis and therapeutic intervention of the HIV epidemic requires understanding the mechanisms by which newly assembled HIV-1 virions are released from infected cells. This knowledge may prove diagnostic in identifying unsuspected sites for intervention and plausible strategies for targeted drug delivery. It is imperative, urgent, and ethical to uncover reliable and selective inhibitors of progeny virus release [1].

Vpu, an accessory membrane protein from the AIDS-associated virus HIV-1 [2], folds into two distinct structural domains with different biological activities: a transmembrane (TM) α-helical domain involved in the budding of new virions from infected cells, and a cytoplasmic domain encompassing two amphipathic helices, which is implicated in CD4 degradation [3],[4]. Without Vpu, the HIV-1 virus remains attached to the surface of the human cell in which it has replicated. This activity of Vpu requires an intact TM α-helical domain [5],[6]. And, it is known that oligomerization of the Vpu TM domain results in the formation of sequence-specific, cation-selective channels [6]–[9]. The molecular mechanism by which Vpu facilitates virion release remains unclear [2]. However, the recent discovery that Vpu neutralizes the effect of tetherin (B cell stromal antigen 2, BST-2/HM1.24) has provided insights into the molecular basis of its action [10],[11]. Tetherin is an interferon-induced host cell membrane protein that blocks the release of HIV-1 virions from the cell surface [10],[11]. This finding posits the notion that abrogation of Vpu function with the consequent suppression of new viable virus release from the host cell surface is a plausible therapeutic strategy in the treatment of HIV/AIDS.

## Hypothesis

We propose that it is feasible to increase the effective cell surface density of Vpu-free tetherin by designing transmembrane peptide decoys that target the Vpu TM α-helix [4], [6]–[9] and abort the assembly of tetherin-Vpu heterooligomers. The consequence of this intervention would be to accrue two populations of Vpu in HIV-1-infected cells: one available for complex formation with tetherin and capable of enhancing the release of new virions, and a second population scavenged in heteromeric complexes with the decoys that would inhibit Vpu binding to tetherin and restrict virion release.

TM peptide decoy design involves identification of sequences that would fold in the proximity of the Vpu TM helix and conform complementary matching surfaces to secure tight binding to Vpu and thereby generate unproductive, membrane-embedded heteromers. It requires exploring the TM sequence landscape to identify optimal Vpu TM matchmakers that would self-assemble into unproductive heterooligomers, thereby overcoming the restrain on tetherin and the consequent restriction of virion release from infected cells. A more refined design would entail identifying sequences that effectively embody intrinsically unfolded conformers [12] that on contact with the Vpu TM adopt secondary structure and bind to complementary surfaces, rendering Vpu unavailable for binding to tetherin. The TM decoys are designed to bury their internal complexity and interact with other TM modules through simple interfaces by surface matching. Fold design must be compatible with the environment to afford its intended function. For membrane proteins, such interfaces must necessarily be complementary with and adjusted to both the hydrophobic core of the lipid bilayer and to the amphipathic character prevalent at the bilayer–water interfaces. A vast number of sequences may underlie such design and be compatible with the bilayer constrains; the challenge is identification.

## Rationale

Tetherin [10],[13] and Vpu [3],[4],[6],[14] exhibit a strong propensity to oligomerize. It is precisely this proclivity which constitutes the essence of the self-assembly of the Vpu channel [15] and of the Vpu–tetherin heterooligomers that underlies Vpu-dependent virion release [10],[11]. At the root is the question of coupling, namely how the TM modules of Vpu and tetherin self-assemble into stable heteromers. The structural underpinning of such assembly is the TM region of Vpu and tetherin. These two proteins may represent a limit of simplicity: a single TM α-helix per polypeptide with an inherent propensity to oligomerize. The beauty of this essential design resides in the fact that both channel activity and virion release activity are thermodynamic consequences of the natural tendency of the membrane-embedded TM helix to aggregate in order to optimize its interactions within the hydrophobic interior of the lipid bilayer, thereby generating a helical bundle, i.e., the structural blueprint for the channel [15],[16] and the Vpu–tetherin heterooligomer [10],[11]. Vpu TM matchmakers would consequently abort the Vpu–tetherin partnership by co-opting the match, converging on a common mechanism.

The rationale underlying this hypothesis is strengthened by a number of additional facts: (1) The intact TM of Vpu is required to enhance virus release [5],[6] and to antagonize the tetherin-dependent retention of nascent enveloped virus [11]. (2) The Vpu TM assembles into tetramers that express ion channel activity which requires an intact TM sequence [7]. (3) The TM of Vpu can be functionally substituted by the TM of the M2 proton channel of influenza virus and yield a pathogenic virus [17]. The implication is that the M2 TM assembles into productive heterooligomers [18],[19] with the Vpu TM and thereby promotes retention of virions attached to tetherin. (4) Overexpression of TASK-1, an acid-sensitive K^+^ channel, markedly impairs the ability of Vpu to enhance virion release [20]. The implication is that the high sequence similarity of Vpu and TASK-1 TMs promotes the assembly of unproductive heterooligomers, thereby rendering Vpu incompetent in virion release. (5) A 20-residue peptide corresponding to the C-proximal region of α1-antitrypsin blocks HIV-1 entry by interacting with the gp41 fusion peptide [21]. The binary complex is associated primarily by hydrophobic interactions between complementary matching surfaces. Small molecules from “credit card” libraries inhibit gp41-mediated cell fusion by disrupting protein–protein interactions underlying the gp41 six-helix bundle formation [22],[23]. These findings lend credence to the hypothesis that short peptide segments derived from the TM of tetherin or similar TM sequences, and small hydrophobic compounds targeted to the Vpu TM, act as Vpu inhibitors by binding to complementary surfaces, thereby supporting the retention by tetherin of newly assembled virions at cell surfaces.

**Figure 1:**
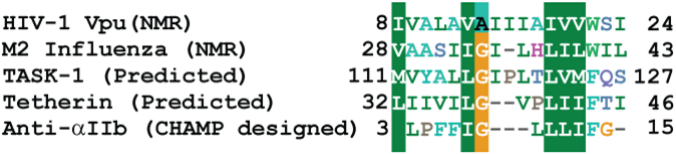
Identification Vpu TM Matchmakers by Sequence Analysis (http://www.ncbi.nih.gov). Selected sequences are displayed (FASTA) and aligned using ClustalW. A single letter code for the amino acid residues is used. Numbers denote residue position in the corresponding sequence. Residue similarity is shaded using the PAM250 substitution matrix appropriate for identifying conserved residues in the alignment of evolutionary distant sequences. Accession numbers are as follows: HIV-1 Vpu, Swiss-Prot entry P05921; M2 influenza, gi 58531181; TASK-1, gi 82542571; BST-2/HM1.24/Tetherin, gi 4757876; anti-αII, designed protein (see [26]).

This hypothesis naturally leads us to outline an initial itinerary to identify the minimal sequence determinants for the folding and coupling between decoy and Vpu TM modules that would minimally entail the following: (1) Identification of Vpu TM matchmakers by sequence analysis. The selection criteria consider experimentally determined TM α-helices or predicted single TM spans with a high propensity to fold into α-helices, and a high sequence similarity to the Vpu TM α-helix. A truncated alignment of relevant TMs with the target sequence of Vpu TM α-helix, as determined by NMR spectroscopy [4],[9],[24] is illustrated in Figure 1. The TM of the M2 proton channel of influenza A virus was selected using the NMR [19] and crystal [18] structures. The TM segments of TASK-1 [20] and tetherin [10],[11],[25] are based on secondary structure predictions. The anti-αIIb sequence is an optimized result obtained using the analysis dubbed CHAMP (computed helical anti-membrane protein), a computational approach to design peptides that specifically recognize the TM helices of natural proteins in a sequence-specific mode [26]. This alignment is remarkable insofar as it highlights the occurrence of a conserved sequence motif compatible with a propensity for intramembrane self-assembly, a requirement for Vpu TM matchmakers. (2) Implementation of the CHAMP [26] analysis combined with integration of the symmetry principles emerging from designed protein–protein associations [27] and the aid of computational refinement focusing on positions of side-chains for favorable pairwise interactions. This should provide a robust method to identify optimal Vpu matchmaker decoys. (3) Structure-based design of additional peptide decoy sequences. The atomic structures of the TM α-helix of Vpu [4],[9],[15],[24] and of the influenza M2 proton channel [18],[19] provide blueprints to examine their critical interactions and should accelerate the design process. This strategy is not limited to the use of a single peptide; combinations may be superior and, ultimately, a cocktail of peptides could be the target to develop. (4) Assessment of the specificity of the design, including the evaluation of control peptide decoy sequences consisting of the same amino acid composition as the test decoy peptides with randomized sequence [6],[11]. (5) An approach that is not limited to the use of peptides. Peptidomimetics are a valid option, as shown by the discovery of a blocker of the N-methyl-D-aspartate (NMDA) receptor channel using combinatorial libraries [28]. (6) Small molecule “credit card” libraries. These are akin to credit cards in terms of being planar, aromatic core structures decorated with chemical diversity that confers on them the feature of inserting into crevices at the interacting surfaces, thereby disrupting the complementarity that underlies the specificity of association [22],[23]. (7) Peptides that could be readily generated by solid-phase methods [6],[24], and circular dichroism used to assess α-helical content [29]. (8) Lead compounds that could be validated based on structure-activity relationships assessing their inhibitory activity on the Vpu-enhanced progeny virus release from CD4^+^ human T cell line A3.01 or HeLa cells infected with HIV-1 clone NL4-3 [5],[6],[11],[30]. Optimal candidates would suggest structural and chemical scaffolds to be used as blueprints for refinement.

## Concluding Remarks

The guiding principle underpinning this hypothesis outlines a new way of thinking about disrupting protein–protein interactions by focusing on the TM anchors of the partners, a hitherto unexplored path towards the development of more effective blockers of virus release that may generate novel leads that could be developed into specific, realistic, and effective medications.
